# Book Review: Counseling: A Comprehensive Profession

**DOI:** 10.3389/fpsyg.2021.704413

**Published:** 2021-06-11

**Authors:** Irene Khosla

**Affiliations:** Discipline of Psychology, School of Social Sciences, Indira Gandhi National Open University, New Delhi, India

**Keywords:** counseling psychology, counselor, skills & competencies, Indian culture, counseling process, ethics & professionalism, theories of counseling

“..I can't always be, what you see in your mind” writes Gladding (1973) as he poeticizes the counseling process. “Counseling is a complex riddle” he says, and rightly so, minimizing the gap between the complex principles and theoretical conceptualizations of counseling and its application in reality is easier said than done.

Acting as a stepping stone to bridge this gap is the book “Counseling: A Comprehensive Profession” (2017) authored by Samuel T. Gladding (Gladding, 2017). A professor of counseling at Wake Forest University and a practicing counselor for over 40 years, the book is a fruitful and effortful product of his immeasurable professional experiences and laborious research and observations.

The book reviewed at present is the Indian edition of “Counseling: A comprehensive profession,” 7th edition, published by Pearson India Education Services in 2017, with inputs from Dr. Renu Kishore, Professor, University of Delhi, covering the various facets of counseling in India. The book contains 20 chapters divided into four parts.

Fastidious and extensive, it covers a broad range of topics. The underlying theme of the book is that “counseling is both a generic and specialized part of the helping profession.” It is noteworthy that the book focuses on the past, the present and the future of counseling, providing a holistic description of not just the background and process of counseling, but also of its applications, implications and ongoing trends. Unlike others, Gladding's book pays significant emphasis to the “persons” involved in counseling, whether focusing on the counselors' skills or the clients background. In addition, the author also sheds light on enhancing sensitivity and professionalism by lengthily discussing about working with multicultural and diverse populations, ethical considerations, and the legal aspects of counseling.

Broadening the scope further to enhance cultural relevance, the Indian edition of the book incorporates the counseling scenario in India. Additions have been made to all chapters. From its manifestation to the present state of counseling in India, cultural integration, Indian laws and ethics, educational, and training requirements and the application of counseling in the Indian context along with statistical and empirical data have been discussed. Case studies have also been modified to match the Indian framework, making it beneficial and relatable for the Indian audience.

Part I of the book has a multidimensional focus. From its conceptualization to the growth and development of the field, this part highlights the competencies and characteristics of counselors while stressing the importance of qualifications and ethical guidelines. Challenges faced in the profession are also addressed here. It is divided into five chapters which illustrate the historical roots and trends in counseling; individual and professional facets of counseling; ethical and legal considerations; working in a multicultural society; and dealing with diversity. Chapter two for example deals with the education and training of counselors (in general and in the Indian context) and their skills, whereas chapter four deals with overcoming the challenges of multicultural counseling.

Part II elucidates the fundamentals, describing how a helping relationship is formed, viewed and how it progresses, and further highlights the processes and theories of various counseling perspectives. So topics such as interviewing and rapport (chapter six), the need for follow up and referral (chapter eight), assumptions of humanistic view (Chapter nine) pros and cons of solution-focused therapy (chapter ten) are discussed here. Of the five chapters in this section, the first three center on building counseling relationships; working relationship; termination; while the last two chapters describe the counseling approaches. For each theory/perspective, the author has put forth a list of strengths and weaknesses along with a mention of their use in India, ensuring to inclusivity.

Part III of the book has four chapters which begin from chapter 11 till chapter 14 wherein the “core counseling” activities are presented including counseling in groups; consultation; research and evaluation; and assessment and diagnosis. For instance, the types and characteristics of groups are described in this part, followed by the concept of consultation and how it differs from counseling. Further, the assessment tools and applicable statistical measures are greatly discussed in this section, with a special mention of tools used in the Indian setting.

Part IV deals with working with “special populations” where the counseling skills and practices are distinguished and individualized based on their purpose and applicability. In this section, the various subfields of counseling are discussed, weighing in on the specialized proficiencies and processes attached to each. With a total of six chapters—career counseling over the life span; family, couple and marriage counseling; school counseling; student life and college counseling; abuse, addiction and disability counseling; and private counseling practices are examined in part four.

In terms of the structure of the book, each chapter begins with an overview of the proceeding topic and is followed by a few “points to think about,” which foster curiosity and interest in the reader. Subsequently, giving the book a personal touch, each chapter is also gracefully introduced with a content specific poem written by the author based on his experiences. All topics mentioned are covered extensively and meticulously, presented in a concise, point-wise style with subtopics put forth clearly. The language is simple and direct so the content is easy to comprehend and imbibe. Questions for personal reflection, summary boxes, tables, diagrams, and images presented in all chapters serve as advantageous and valuable for gaining conceptual clarity. Case examples are given throughout the book which aid in relating theories to practical relevance. All chapters are summarized and concluded in the end. Appendix A of the book is particularly informative and resourceful as it cites various counseling organizations active in the United States of America and India. Overall, the book is proficient in keeping the reader engrossed.

In conclusion, the book is exhaustive with a multidimensional focus on the ever so dynamic and complex field of counseling. The amalgamation of theoretical concepts with realistic examples and cultural relatedness makes the book highly pertinent for students and for anyone willing to venture into the counseling profession. So just like a building cannot stand without a strong foundation, a counselor cannot exist without building their repositories of knowledge, about what counseling truly is and how a counselor works. Thus, the book is a stupendous means to gain cognizance and expertise in the field of counseling.

## Author Contributions

The author confirms being the sole contributor of this work and has approved it for publication.

## Conflict of Interest

The author declares that the research was conducted in the absence of any commercial or financial relationships that could be construed as a potential conflict of interest.

## References

[B1] Gladding S. T. (1973). Reality sits in a green-cushioned chair. Pers. Guid. J. 52, 224–224.

[B2] Gladding S. T. (2017) Counseling: A Comprehensive Profession, (7th Edn). Noida: Pearson Education India.

